# A Recurrent Deep Network for Estimating the Pose of Real Indoor Images from Synthetic Image Sequences

**DOI:** 10.3390/s20195492

**Published:** 2020-09-25

**Authors:** Debaditya Acharya, Sesa Singha Roy, Kourosh Khoshelham, Stephan Winter

**Affiliations:** 1 Department of Infrastructure Engineering, The University of Melbourne, Parkville, Victoria 3010, Australia; k.khoshelham@unimelb.edu.au (K.K.); winter@unimelb.edu.au (S.W.); 2 Department of Manufacturing, Materials and Mechatronics, RMIT University, Carlton, Victoria 3053, Australia; 3 Institute for Sustainable Industries and Livable Cities, Victoria University, Werribee, Victoria 3030, Australia; sesa.singharoy@live.vu.edu.au

**Keywords:** indoor localisation, camera pose regression, 3D building models, long short term memory

## Abstract

Recently, deep convolutional neural networks (CNN) have become popular for indoor visual localisation, where the networks learn to regress the camera pose from images directly. However, these approaches perform a 3D image-based reconstruction of the indoor spaces beforehand to determine camera poses, which is a challenge for large indoor spaces. Synthetic images derived from 3D indoor models have been used to eliminate the requirement of 3D reconstruction. A limitation of the approach is the low accuracy that occurs as a result of estimating the pose of each image frame independently. In this article, a visual localisation approach is proposed that exploits the spatio-temporal information from synthetic image sequences to improve localisation accuracy. A deep Bayesian recurrent CNN is fine-tuned using synthetic image sequences obtained from a building information model (BIM) to regress the pose of real image sequences. The results of the experiments indicate that the proposed approach estimates a smoother trajectory with smaller inter-frame error as compared to existing methods. The achievable accuracy with the proposed approach is 1.6 m, which is an improvement of approximately thirty per cent compared to the existing approaches. A Keras implementation can be found in our Github repository.

## 1. Introduction

Deep convolutional neural networks (CNNs) have been successfully used to perform localisation using single images without the need of an initial location [1,2,3,4]. These approaches consist of regressing the camera pose (location and rotation) of a single image with deep CNNs that have been fine-tuned using labelled real images. The labels (known poses) are generated from a 3D reconstruction of the indoor space using images, which is usually performed using the structure-from-motion (SfM) approach [5]. The SfM approach involves creating a database of overlapping images of the whole indoor space, which presents challenges for large indoor environments. This challenge limits the wide applicability of the CNN-based camera pose regression approaches [6].

Building information model (BIM)-PoseNet [7] and Bayesian BIM-PoseNet [8] eliminate the requirement of 3D image-based reconstruction using a texture-less 3D model of the indoor space. These approaches fine-tune a deep CNN using synthetically rendered images from a building information model (BIM), to estimate the pose of real images. However, the camera pose for each image frame is estimated without considering the spatial dependency between the frames. The per-frame error varies largely, thus resulting in high variance of distances between two consecutive frames.

Therefore, a relevant question is whether the performance of approaches that use synthetic images for visual localisation, such as [7,8], can be improved by incorporating spatio-temporal constraint between consecutive images. In addition, the uncertainty of camera pose plays a vital role in the estimation, as it provides the confidence of the estimations in the absence of ground-truth. Ideally, localisation errors should be correlated with the estimated uncertainties. The uncertainty of camera pose has been modelled using synthetic images [8]. However, that approach estimates the localisation uncertainty from a single image without considering the spatio-temporal continuity of image sequences. The pose estimates vary largely for consecutive images resulting in a jagged trajectory. Therefore, we explore another relevant question as to whether the uncertainty of camera pose can be modelled by using a sequence of synthetic images.

In this article, we propose Recurrent BIM-PoseNet (A Keras implementation can be found at https://github.com/debaditya-unimelb/RecurrentBIM-PoseNet), a deep Bayesian recurrent CNN that utilises synthetic image sequences obtained from a BIM, thereby eliminating the requirement of 3D reconstruction of the indoor spaces. At the test time, the camera poses of a sequence of real image is regressed. Our method takes advantage of temporal dependencies between consecutive images in a sequence to model the uncertainty, reduce the pose estimation error and generate a smoother trajectory. By smoothness, we mean having consistent inter-frame distances so that there are no sudden jumps between two consecutive camera poses.

We show that the pose estimations by Recurrent BIM-PoseNet result in a smoother trajectory with smaller inter-frame errors as compared to the state-of-the-art approaches. Additionally, the estimated uncertainties show correlation with the localisation errors, suggesting the suitability of the approach for modelling uncertainty. Moreover, we show that the proposed method does not need any colour or texture information to perform localisation. The main contributions are:

1. We improve the localisation accuracy of pose regression networks that use synthetic images to estimate the camera pose of real images. The spatio-temporal constraint of image sequences is utilised to improve accuracy and to generate a smoother trajectory.
2. The uncertainty of camera pose estimation is modelled by sampling from a sliding window of image sequences. We show that the modelled uncertainty shows correlation with the errors.

Section 2 reviews the visual approaches to indoor localisation. In Section 3, the theory and methodology are explained, which is followed by experiments and results in Section 4. Section 5 concludes the outcomes of the research and the possible future directions.

## 2. Background and Related Work

The primary limitation of many computer vision approaches, such as SLAM [9], visual odometry [10] and 3D model-based tracking [11] is the requirement of an initial location. This initial location is often derived from image-based retrieval approaches. The image-based retrieval approaches that provide the initial camera pose can be classified into three categories [12].

### 2.1. Image-Based Retrieval Approaches

#### 2.1.1. Using Point Features

The first category includes approaches (such as [13]) that match the point features with an existing database of features, like 3D point clouds. These approaches estimate the camera pose directly using classical photogrammetry techniques like P3P [14], where the 3D information of each point feature is retrieved from the point clouds. The main limiting factor for these approaches is the dependency on the point clouds that are usually generated from SfM approaches, thus requiring a 3D reconstruction of the indoor space in advance to localisation.

#### 2.1.2. Depth-Based Approaches

The second category includes depth-based approaches [15,16]. These approaches use depth cameras (or RGB-D cameras) to assign a location to each pixel of the image by comparing with a pre-existing depth map [15]. Cavallari et al. [17] adapt offline-trained regression forests to regress location in new indoor spaces, by dynamically updating the learnt model from a few training examples of the new space. Brachmann et al. [18] propose a differentiable RANSAC (DSAC) framework that uses RGB-D images to train a CNN that predicts the scene coordinates. Subsequently, the DSAC pipeline was optimised resulting in DSAC++ where state-of-the-art accuracies are reported [16]. These approaches depend on the 3D depth map of the indoor space from depth cameras, thus limiting their applicability only with depth cameras.

#### 2.1.3. CNN-Based Approaches

The third category includes approaches that use deep CNNs for single image camera pose regression, like PoseNet [1]. These approaches fine-tune pre-trained networks using annotated real images obtained from SfM approaches to estimate the camera pose of an input image. The works of [3,19] improve the pose regression accuracy with a new geometric loss function and data augmentation method, respectively. Walch et al. [4] propose the use of a CNN-LSTM architecture to perform a structural dimensionality reduction of image features derived from the CNN to improve accuracy. Uncertainty has been modelled for CNN pose regression with Bayesian PoseNet [2] where dropout is used to draw Monte Carlo samples. The CNN-based pose regression approaches estimate the camera pose independently and do not exploit the valuable constraint of spatio-temporal smoothness. The pose estimates of such approaches lead to cases where the inter-frame distances are larger than the camera motion. Clark et al. [20] propose VidLoc, a recurrent network architecture to smooth the estimated trajectory and model the uncertainty of camera pose estimates.

The major limitation of using the deep CNN for camera pose regression is the requirement of large number of annotated real images. In practice, capturing thousands of overlapping images and estimating their corresponding poses by SfM approaches is challenging for large indoor environments. A potential solution to eliminate this requirement it to use photo-realistic synthetic images generated from an available 3D model.

### 2.2. Use of Synthetic Images

Jian et al. [19] use a 3D model reconstructed from real images to improve the camera pose regression, by generating synthetic images. A coarse visual localisation is performed using images and a BIM [21], where the authors compare the real and the synthetic images on the basis of features obtained from a CNN using cosine similarity. The authors classify the real image to its nearest synthetic image of known location and orientation.

BIM-PoseNet [7] utilises synthetic images obtained from a texture-less 3D model to train a network that estimates the camera poses of real images. The authors achieve an accuracy under 2 metres by representing the real and the synthetic images as edge gradient magnitude (gradmag). Subsequently, the uncertainty of pose estimation of real images was modelled using synthetic images by Bayesian BIM-PoseNet [8]. However, the estimated camera poses of these approaches are less precise, compared to the approaches using real images. Further, the estimated poses show a high variation between consecutive images due to the missing spatio-temporal constraint between the consecutive images.

### 2.3. Limitations of Current Approaches

A limitation of [13,19,20] is the requirement of 3D reconstruction by SfM methods. The drawback of the works of [17,18] is the dependency on RGB-D cameras, thus deeming it unsuitable for most of the smartphone cameras. The shortcoming of the work by [21] is the coarse localisation, and the inability to interpolate the absolute camera pose. Lastly, the works of [7,8] suffer from high variance of distances between consecutive frames.

In contrast, we do not perform 3D reconstruction by SfM methods, rather utilise synthetic images rendered from a texture-less 3D building model to adapt to new indoor scenes. Moreover, our approach can interpolate the absolute camera pose effectively in space between the synthetic training frames by regression, instead of predicting the nearest known location of the synthetic images. Lastly, compared to [7,8], we reduce the errors and generate a smoother trajectory.

In VidLoc [20], the authors drop the fully connected layers to improve the inference time of the network, to compensate for using multiple frames. Moreover, the authors performed all the experiments for real images only. In contrast, we retain the fully connected layers and demonstrate the advantage both for the real-real (fine-tuned with real and tested on synthetic) and synthetic-real (fine-tuned with synthetic and tested on real) cases. The presence of fully connected layer improves the camera pose estimation, especially for the synthetic-real case.

## 3. Methodology

**Figure 1:**
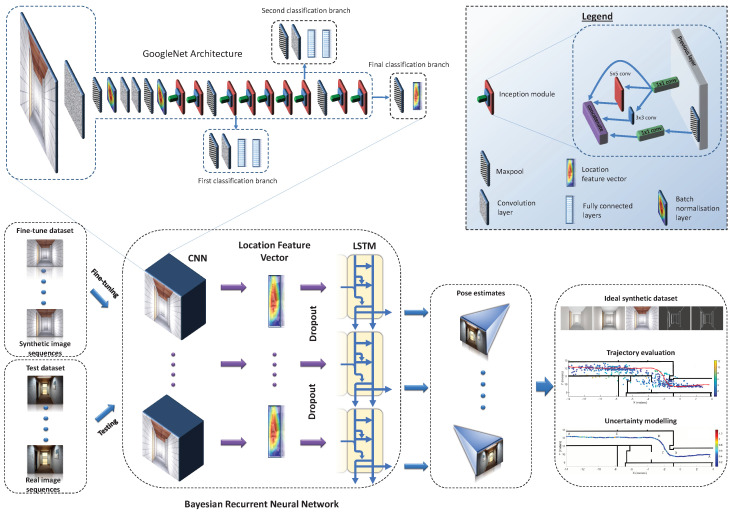
The design of the proposed approach. The network used is GoogleNet [22] containing nine inception modules. The output of the final classification branch yields the location feature vector which serves as input to the LSTM layer.

The design of the proposed approach is shown in Figure 1. Recurrent BIM-PoseNet is fine-tuned using several types of synthetic image sequences generated from a 3D indoor model or a BIM. Subsequently, the networks are tested using real image sequences captured by a smartphone camera. The network regresses the camera poses corresponding to each test image in the sequence. The generation of the synthetic image sequences is described in Section 3.1. The architecture of Recurrent BIM-PoseNet is described in Section 3.2, fine-tuning and loss function are described in Section 3.3, and Section 3.4 explains the uncertainty modelling for camera pose estimations. The implementation details are presented in Section 4.1.

### 3.1. Generation of Synthetic Image Sequences

Synthetic images (The synthetic and the real datasets with their corresponding ground-truth poses are available at https://melbourne.figshare.com/articles/UnimelbCorridorSynthetic_zip/10930457) are obtained by rendering from a 3D model using different texture and lighting options. Previous work [7] has shown that a network fine-tuned using edge rendering (i.e., without texture and lighting) can successfully estimate the pose of real images represented as edge gradient magnitude (gradmag). Other types of rendering, such as the Cartoonish, photo-realistic and photo-realistic textured can also be used for the localisation task. Therefore, the first relevant question is which type of synthetic image is most suitable for the task of localisation with Recurrent BIM-PoseNet. Hence, the current research proposes to use several types of synthetic image sequences derived from the 3D indoor model to fine-tune Recurrent BIM-PoseNet and test using real image sequences.

**Figure 2:**
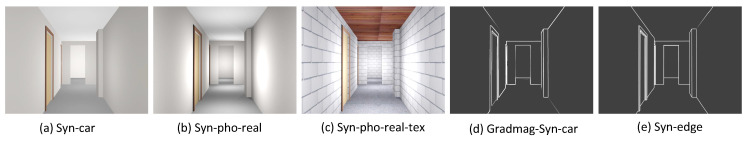
The five types of synthetic images generated from the 3D indoor model, after Acharya et al. [7].

Five sets of synthetic image sequences were generated, namely Cartoonish (Syn-car), photo-realistic (Syn-pho-real), photo-realistic textured (Syn-pho-real-tex), Gradient magnitude of Cartoonish (Gradmag-Syn-car) and Edge render (Syn-edge) with their respective ground-truth poses, as shown in Figure 2. The name in the bracket represents the pseudonyms of the respective datasets for convenience, and the naming convention is according to Blender (Blender is an open-source 3D computer graphics software that is used to perform simulations and animated films. Visit www.blender.org for more information.), which is used to render the synthetic images.

Syn-car images (Figure 2a) were generated using a rendering model that roughly traces the path of the light. Syn-pho-real (Figure 2b) images were generated using an advanced light tracing model that follows the physical rules of light scattering and reflection. Syn-pho-real-tex (Figure 2c) images are also generated using the same light tracing model as Syn-pho-real. However, these images contain the synthetic texture of objects like brick and carpet texture on the walls and floor, respectively. Gradmag-Syn-car (Figure 2d) images are derived from Syn-car images by taking gradient magnitude of the images. Edge render images (Figure 2e) were generated by rendering only the edges of the 3D indoor model in the Field-of-View (FoV) of the virtual camera. More details of the synthetic dataset is present in Section 4.3.

### 3.2. Deep Learning Architecture

The proposed network consists of a deep Bayesian CNN and an LSTM layer to capture the spatio-temporal dependencies of consecutive frames. The LSTM [23] is a particular recurrent neural networks that is capable of learning long-term patterns in the input data. A standard LSTM consists of forget, input, output and reset gates in addition to a memory cell that enables the flow of data in and out of the memory cells that is regulated by the input and forget gates. We use GoogleNet [24] for image feature extraction by removing the softmax layers, and adding a 2048-dimensional dense layer (location feature vector). GoogleNet pre-trained on the Places image dataset [25] are used as the starting weights due to its suitability for the task of scene classification [1].

In the literature, sliding window has been used to generate samples from the data. These samples have been used by the LSTMs to exploit the spatio-temporal information of consecutive samples, and subsequently to model the uncertainty [20,26]. Therefore, to capture the spatio-temporal information, we generate sequences of images by sliding a window of length *n*, from the total number of *N* images. The sequences are the input to the network. The CNNs generate a sequence of location feature vectors ($v_{1},v_{2},\ldots ,v_{n}$) which is the input to the LSTM layer (refer to Figure 1). The output of the hidden states of the LSTM is utilised to estimate the pose of the sequence of images.

### 3.3. Fine-Tuning and Loss Function

The output of the LSTM units are connected to a 7-dimensional dense layer to regress *n* camera pose estimates ($p_{t}^{T}$) at each time step, which is defined as:

(1) $$p_{t}^{T}=\left[x_{t}^{T},q_{t}^{T}\right]$$

where $x_{t}^{T}$ is the 3-dimensional location vector and $q_{t}^{T}$ is the 4-dimensional rotational vector of the camera in quaternion format, for $T^{\mathrm{th}}$ sequence for time step *t*.

To add the spatio-temporal constraint to the camera pose estimations during the training procedure, a loss function *L* (Equation (2)) was used that represents the errors of the “whole” sequence of images by unrolling the network and performing back-propagation through time. This constraint enabled the network to learn to estimate consistent camera poses for the test image sequences.

(2) $$L=\sum_{t=1}^{t=n}\left|\right|{\hat{x}}_{t}^{T}-x_{t}^{T}{\left|\right|}_{2}+\beta ||{\hat{q}}_{t}^{T}-q_{t}^{T}{\left|\right|}_{2}$$

where ${\hat{x}}_{t}^{T}$ and ${\hat{q}}_{t}^{T}$ are the estimated values of location and rotation vector of the $t^{\mathrm{th}}$ image of the sequence and β is the scaling factor to weigh the location and the rotational errors, respectively. The value of β is dependent on the indoor scene and is determined experimentally.

### 3.4. Modelling Uncertainty

During the test phase, the network regresses the unknown pose of the images from a window of real images. The uncertainty is modelled by gathering posterior distribution of the weights of the network [27]. The Monte Carlo samples are obtained by applying dropout during testing phase on the output of the network, and the mean of the samples is considered as the pose estimate. We use the trace of the covariance matrices of the camera pose samples, which provides a good numeric measure of the uncertainty. The multiple pose predictions by the network are utilised in the following manner to model the location and rotation uncertainties:

(3) $$\begin{array}{c} \begin{array}{c} U_{x_{t}}^{T}=\mathrm{sqrt}\left(\mathrm{trace}\;\left(C\;\left(x_{1}^{T},x_{2}^{T},\ldots ,x_{n}^{T}\right)\right)\right),\;U_{q_{t}}^{T}=\mathrm{sqrt}\left(\mathrm{trace}\;\left(C\;\left(q_{1}^{T},q_{2}^{T},\ldots ,q_{n}^{T}\right)\right)\right) \end{array} \end{array}$$

where, $U_{x_{t}}^{T}$ and $U_{q_{t}}^{T}$ denote the uncertainty of camera location and rotation, respectively for the $T^{\mathrm{th}}$ frame, and *C* denotes the covariance matrix. Subsequently, the correlation factor R [8] is defined to quantify the correlation of the estimated localisation uncertainty and localisation errors in the following equations:

(4) $$\begin{array}{c} \begin{array}{c} R_{LU/LE}=\frac{\mathrm{cov}(U_{x}^{N},E_{x}^{N})}{\sigma_{U_{x}^{N}}\sigma_{E_{x}^{N}}},\;R_{RU/RE}=\frac{\mathrm{cov}(U_{q}^{N},E_{q}^{N})}{\sigma_{U_{q}^{N}}\sigma_{E_{q}^{N}}},\;R_{LU/RU}=\frac{\mathrm{cov}(U_{x}^{N},U_{q}^{N})}{\sigma_{U_{x}^{N}}\sigma_{U_{q}^{N}}} \end{array} \end{array}$$

where $R_{LU/LE}$ denotes the correlation of estimated location uncertainties vs. location errors, $R_{RU/RE}$ denotes the correlation of the estimated rotation uncertainties vs. rotation errors and $R_{LU/RU}$ denotes the correlation of the estimated location uncertainties vs. estimated rotation uncertainties. $U_{x}^{N}=\left[U_{x_{t}}^{1},U_{x_{t}}^{2},\ldots .,U_{x_{t}}^{N}\right]$ is the vector containing the estimated location uncertainties, $U_{q}^{N}=\left[U_{q_{t}}^{1},U_{q_{t}}^{2},\ldots .,U_{q_{t}}^{N}\right]$ is the vector containing the estimated rotation uncertainties, $E_{x}^{N}=\left[E_{x_{t}}^{1},E_{x_{t}}^{2},\ldots .,E_{x_{t}}^{N}\right]$ is the vector containing the location errors and $E_{q}^{N}=\left[E_{q_{t}}^{1},E_{q_{t}}^{2},\ldots .,E_{q_{t}}^{N}\right]$ is the vector containing the rotation errors. $\sigma_{U_{x}^{N}}$, $\sigma_{U_{q}^{N}}$, $\sigma_{E_{x}^{N}}$ and $\sigma_{E_{q}^{N}}$ denote the standard deviations of $U_{x}^{N}$, $U_{q}^{N}$, $E_{x}^{N}$ and $E_{q}^{N}$, respectively, and cov represents the covariance between two random variables.

## 4. Experiments and Results

### 4.1. Implementation Details

The networks were implemented in Keras [28] using TensorFlow libraries [29] on Linux. Adam gradient descent optimisation algorithm with a fixed learning rate of $10^{-3}$ was used to fine-tune the networks for 400 epochs. A Tesla P100 graphics processor unit (GPU) was used to accelerate the code with NVIDIA CUDA^®^ Deep Neural Network library (cuDNN). The fine-tuning and testing images were resized to a resolution of 320 × 240 pixels, and a central crop of dimension 224 × 224 was applied. The mean of the fine-tuning dataset was subtracted from the input images during fine-tuning and test time.

### 4.2. Experimental Design

To evaluate the performance of Recurrent BIM-PoseNet and determine which rendering of synthetic images is the most suitable for camera pose estimation and modelling uncertainty, we design the following experiments.

- Experiment 1: Using real images for training and testing. A baseline accuracy was established by fine-tuning Recurrent BIM-PoseNet using real images, to compare the results obtained from the proposed approach being fine-tuned using synthetic data. Parameters such as ideal LSTM length, ideal window length and the correlation of localisation errors with the estimated localisation uncertainties are identified. The results are presented in Section 4.4.
- Experiment 2: Using synthetic images for training and real images for testing. In this experiment, Recurrent BIM-PoseNet was fine-tuned using several types of synthetic image sequences. Subsequently, pose regression ability of these fine-tuned networks were evaluated by using real image sequences during test, and compared with the previous approaches. The results are presented in Section 4.5.
- Experiment 3: Modelling uncertainty. This experiment consisted of modelling the uncertainty of the estimated camera poses, and evaluating the correlation of the localisation errors with the estimated localisation uncertainties. The results are compared with the results of Bayesian BIM-PoseNet, and are presented in Section 4.6.

### 4.3. Dataset

**Synthetic image dataset:** A 3D indoor model was obtained from a BIM that was created as a part of the ISPRS benchmark on Indoor modelling [30], and was used in our previous works [31,32,33]. The BIM consists of the third floor of Block B of the Department of Infrastructure Engineering at the University of Melbourne, Australia and covered a part of the corridor with an area of 230 $m^{2}$. The level-of-detail (LoD) of this BIM can be considered as LoD 300 as per the BIM specifications [34,35].

The synthetic images were generated by moving a virtual camera in the BIM at a spacing of 5 centimetres along a trajectory that is approximately 30 metres long. Additional images were generated by rotating the camera by 10${}^{\circ }$ around the Y and Z axes, to address the problem of the lower number of images for fine-tuning the networks as pointed out by Jian et al. [19]. For each type of rendering, 2500 synthetic images were generated, having a resolution of 640×480 pixels.

**Figure 3:**
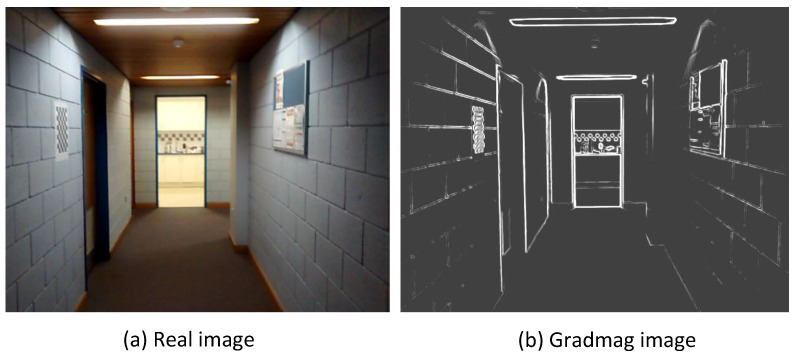
(**a**) A real sample image and (**b**) the corresponding gradient magnitude (gradmag) image after thresholding weak edges.

**Real image dataset:** A total number of 950 images having a resolution of 640×480 were captured by a smartphone camera having a FoV approximately equal to 56${}^{\circ }$. The focus of the camera was fixed to avoid any out-of-focus images, and the exposure of the images was fixed to limit underexposed images. The camera was calibrated to estimate the intrinsic parameters that were used to design the virtual camera. However, the distortion of the images was not modelled for the virtual images. The images were acquired at a constant rate of 30 frames per second. The edge images of the real images were generated by taking the gradmag of the images and suppressing the weak edges below a threshold. A sample real image and its corresponding gradmag image are shown show in Figure 3a,b, respectively.

### 4.4. Experiment 1: Baseline Performance Using Real Image Sequences

A baseline accuracy was established using real image sequences with known ground-truth camera poses (see Supplementary Material). We set the value β in the loss function (Equation (2)) as 600 based on our previous experiments [7]. The whole dataset was used to create real image sequences containing consecutive images, which was partitioned randomly into fine-tuning, validation and testing sets (split 60:20:20).

**Achievable accuracy:** The length of the LSTM units is important to the performance of the network. A longer length of LSTM units will lead to over-fitting the network, where the network will perform excellently for the validation data but will perform poorly for other unseen test data. On the other hand, short LSTM units will lead to under-fitting where the network will not be able to perform well on validation and test data as well. Additionally, the input to LSTM units are the location feature vector, having a length of 2048. Therefore the short length of LSTM units might not be able to correlate the image features with the camera pose.

To identify the ideal number of LSTM units required for the proposed network, we performed an experiment by fine-tuning networks with different LSTM lengths (64, 128, 256, 512, 1024 and 2048). The window size for this experiment was maintained at a constant of three images. It was experimentally identified that there is no improvement in the accuracy of the estimations using more than 512 LSTM units. Therefore, for the rest of the experiments using real data, we use 512 LSTM units.

**Figure 4:**
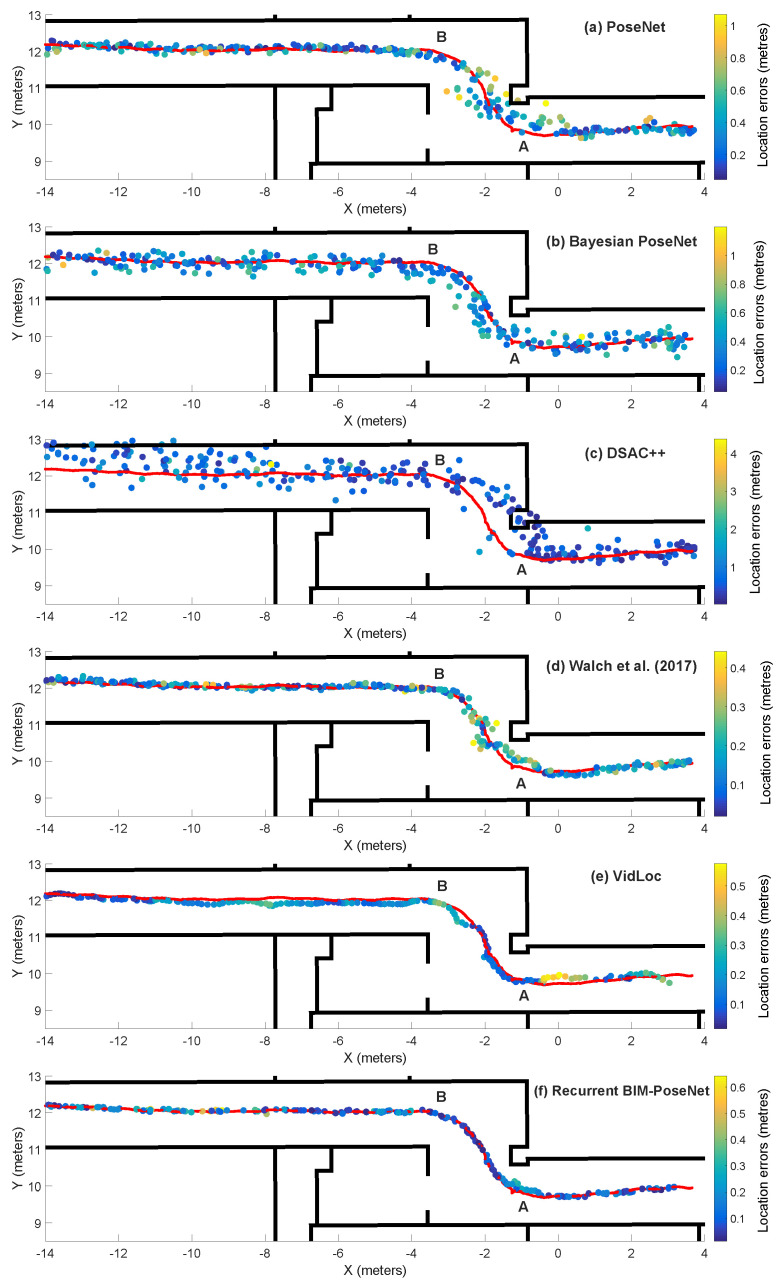
The predictions of (**a**) PoseNet [1], (**b**) Bayesian PoseNet [2], (**c**) differentiable RANSAC (DSAC)++ [16], (**d**) Walch et al. [4], (**e**) VidLoc [20], (**f**) Recurrent building information model (BIM)-PoseNet fine-tuned and tested using a sequence of ten real images. The colour represents the magnitude of error of each point, and the red line denotes the ground-truth trajectory.

To identify the ideal window length, the networks were fine-tuned and tested using different window lengths of images. We fine-tuned four networks using a window of 3, 5, 10 and 15 images and tested them with a window of 1, 3, 5, 10 and 15 images. It was identified that the network fine-tuned using a window of ten image sequences and tested with a window of ten images performs best amongst the other networks, and the median of test error is 0.17 m and 0.83${}^{\circ }$ Figure 4f shows the distribution of the predicted points by the network.

**Comparison with previous approaches:** We compare the proposed approach with PoseNet [1], Bayesian PoseNet [2], DSAC++ [16], our implementation of VidLoc [20] and Walch et al. [4]. Figure 4 shows the estimated trajectories for the different approaches. It is observed that PoseNet, Bayesian PoseNet and DSAC++ lack the temporal smoothness for the whole trajectory, and contains large errors near the turn AB of the corridor which is prone to motion blur. Although the approaches proposed by [4] and VidLoc produce considerably smoother trajectories, they fail to generate consistent results near the turn AB of the trajectory. It is observed that the trajectory predicted by Recurrent BIM-PoseNet is consistent and smoother for the whole trajectory length as compared to the other approaches.

Table 1 summarises the errors and the inter-frame distances for the approaches. It is observed that the errors and the inter-frame distances of PoseNet and Bayesian PoseNet are similar. DSAC++ is known to perform poor for datasets containing a lesser number of training images, and can plausibly explain the poor performance for the current dataset with approximately 600 training images. The approach proposed by [4] performs better than the aforementioned approaches. It is observed that by using a window of ten images, the location and rotation errors for Recurrent BIM-PoseNet approach are reduced by a factor of 3 as compared with PoseNet and Bayesian PoseNet. Although VidLoc and Recurrent BIM-PoseNet show comparable results in terms of inter-frame distances, Recurrent BIM-PoseNet performs better in terms of absolute errors, and demonstrates the advantage of retaining the fully connected layers.

**Figure 5:**
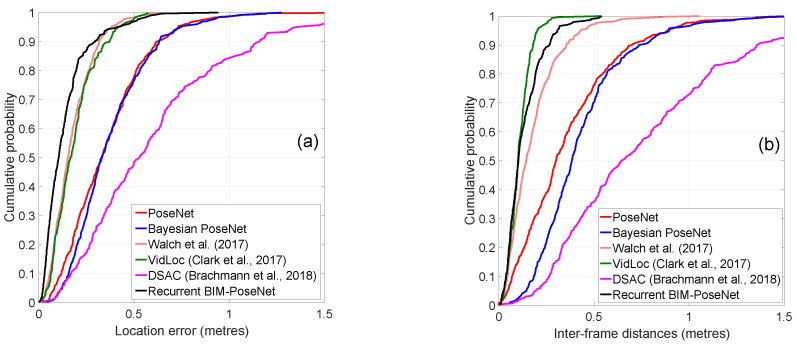
Cumulative distribution function (CDF) plots of the (**a**) errors and (**b**) inter-frame distances of the estimated camera poses for different approaches for Experiment 1.

Figure 5 shows the cumulative distribution function (CDF) of the location errors and the inter-frame distances for the approaches. The location errors are calculated as the Euclidean distances from the ground-truth, and the inter-frame distances are the Euclidean distances between two consecutive frames. Figure 5 shows that Recurrent BIM-PoseNet is the most precise in terms of localisation errors, and its performance is competitive compared to VidLoc in terms of inter-frame distances. The precision of the approach proposed by [4] is also challenging to Recurrent BIM-PoseNet and VidLoc. The performance of PoseNet and Bayesian PoseNet are very close, DSAC++ being the worse performing.

**Figure 6:**
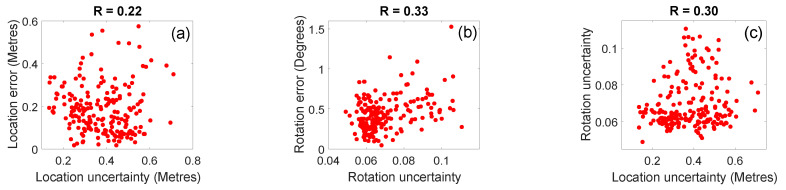
The modelled uncertainty by Recurrent BIM-PoseNet. (**a**) Estimated location uncertainty vs. location errors. (**b**) Estimated rotation uncertainty vs. rotation errors. (**c**) Estimated location uncertainty vs. estimated rotation uncertainty. R denotes the correlation factor.

**Estimated uncertainty:** Figure 6 shows the correlation between the estimated uncertainties and the actual errors for the predictions of a network that was fine-tuned and tested with a window of ten images. Compared to the uncertainty modelled by Bayesian BIM-PoseNet on the same dataset [8], Recurrent BIM-PoseNet shows a better correlation between the estimated location uncertainty vs. location errors (R=0.31), as well as for the estimated rotation uncertainty vs. rotation errors (R=0.67). However, the location uncertainty vs. rotation uncertainty estimated by Recurrent BIM-PoseNet shows low correlation (R=0.22) with the rotation errors as compared to Bayesian BIM-PoseNet.

**Figure 7:**
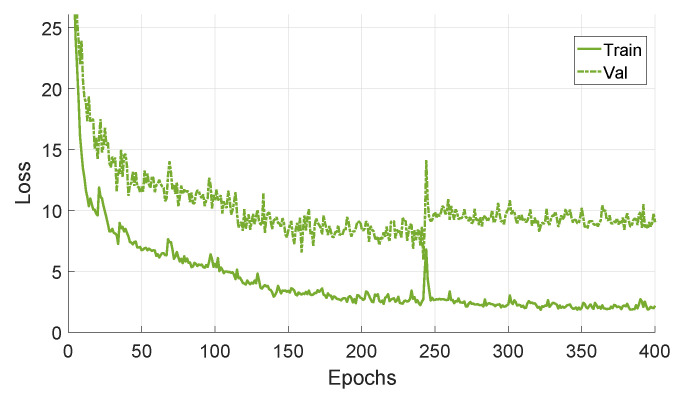
The training and validation losses for the network fine-tuned and validated with real image sequences, having a window size of 10.

Figure 7 shows the training and validation losses for network fine-tuned and validated with real image sequences, with a window of 10 images. The results indicate that the validation loss did not improve after 250 epochs, and the best network was selected as the one having the least validation loss.

### 4.5. Experiment 2: Performance of the Network Fine-Tuned Using Synthetic Images and Tested with Real Images

Recurrent BIM-PoseNet was fine-tuned with the five synthetic image datasets as shown in Figure 2, using β=600, and subsequently, these fine-tuned networks were tested with real images as shown in Figure 3. The networks fine-tuned with images Syn-Car, Syn-photo-real and Syn-Pho-real-tex were tested with the real image (Figure 3a) and the networks fine-tuned with Gradmag-Syn-Car and Syn-edge images were tested with gradmag of the real image (Figure 3b). We used naive (0–0.5) and variational dropouts (0–0.5) for the LSTM units [36] which improved the accuracy of camera pose regression being tested with real images.

Similar to the experiments with real data, to identify the ideal length of the LSTM units for synthetic image sequences, we fine-tune networks using different LSTM lengths (64, 128, 256, 512, 1024 and 2048 units). Gradmag-Syn-car dataset, which has shown the best performance among different renderings [8] was used to fine-tune the networks. For fine-tuning and testing, a window of length three was used. It was identified that there is no improvement in the location and rotation accuracy beyond using 256 LSTM units. Compared to using real data (512 units), this is lower and can be explained by the less information content of synthetic images as compared to real images.

**Figure 8:**
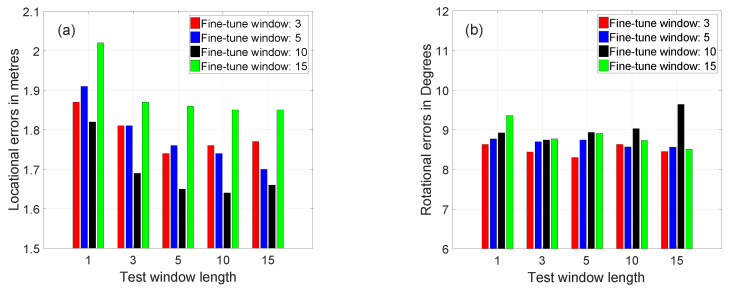
The effects of window length on the (**a**) location errors and (**b**) rotation errors for networks fine-tuned using Gradmag-Syn-car dataset and tested on gradmag of real images.

Figure 8 shows the location and rotation errors for the network that was fine-tuned using different window lengths of Gradmag-Syn-Car dataset and tested with varying window lengths of gradmag of real images. Similar to the benchmark experiment using real images, Figure 8 indicates that the localisation accuracy of the network fine-tuned using a window of ten synthetic images and tested with a sequence of ten real images is the best amongst the other combinations, where the achievable accuracy is 1.62 m and 9.29${}^{\circ }$.

The fine-tuned networks can generalise better on a longer window of test image sequences than when it was fine-tuned. For instance, consider the network that was fine-tuned on a window of three synthetic images, which makes good predictions of the location and rotation of the real image sequences having a window length of 5, 10 and 15 images. The performance of the networks to predict the location of image sequences having a shorter window length is worse, compared to testing with the same window length. For example, consider the network fine-tuned using a window of ten images, where its performance for predicting the location of the sequence of images having a length of 1 and three images is worse, compared to testing it using a window of ten image sequences. In contrast, this trend is not valid for rotational errors, where the networks can make sound predictions of rotation for a longer and shorter window of image sequences than when it was fine-tuned. Figure 8b shows that there is a slight decrease in the rotation errors with the increase in the length of the window of the image sequence.

**Accuracy evaluation and comparison with previous works:** To evaluate and compare the performance using different types of synthetic images, five networks were fine-tuned using the five different synthetic datasets and tested using real images. As the datasets, Syn-car, Syn-pho-real and Syn-pho-real-tex are similar in appearance to the real image, the networks fine-tuned using these datasets were tested directly using real images. However, the networks that were fine-tuned using the gradmag datasets, namely Gradmag-Syn-car and Syn-edge were tested using gradmag of real images. For fine-tuning all the networks, a window of ten synthetic images was used, and for testing a window of ten real images was used.

**Figure 9:**
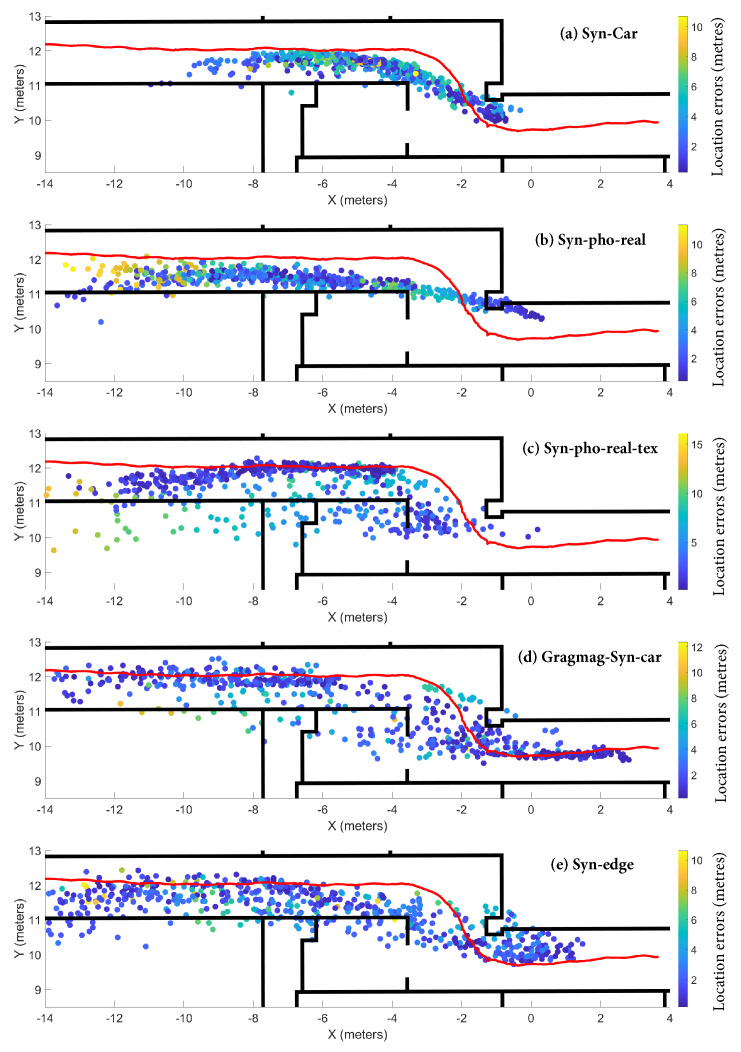
Estimated camera poses by the Recurrent BIM-PoseNet fine-tuned using (**a**) Syn-car, (**b**) Syn-pho-real, (**c**) Syn-pho-real-tex, (**d**) Gradmag-Syn-car, (**e**) Syn-edge. Networks fine-tuned with Syn-Car, Syn-pho-real and Syn-pho-real-tex images were tested with real images, whereas networks fine-tuned with Gradmag-Syn-car and Syn-edge were tested with gradmag of real images.

Figure 9 shows the estimated trajectories by Recurrent BIM-PoseNet fine-tuned with different synthetic image datasets. It is observed that the distribution of the estimated points for the network fine-tuned with Gradmag-Syn-car images (Figure 9d) is more consistent with the ground-truth, and a similar trend is noticed for the Syn-edge images (Figure 9e). Additionally, the bias or shift in the location of the estimated points reported in [7,8] is greatly reduced, especially for the Gradmag-Syn-car dataset. The predictions for the network fine-tuned with Syn-Car, Syn-pho-real and Syn-pho-real-tex are skewed and deviate from the ground-truth.

**Figure 10:**
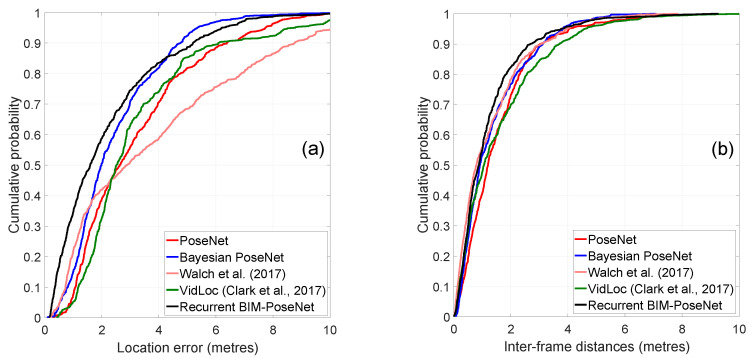
CFD plots of the (**a**) localisation errors and (**b**) inter-frame distances of the estimated camera poses for different approaches for Gradmag-Syn-car dataset.

Figure 10 shows the CDF plots of the localisation errors and the inter-frame distances of the different approaches. The errors and the inter-frame distances are calculated as explained in Experiment 1. It is evident that Recurrent BIM-PoseNet outperforms all the other approaches in terms of precision. The inter-frame distances of all the approaches are comparable; however, only a slight improvement is identified for Recurrent BIM-PoseNet.

Table 2 shows the test errors of Recurrent BIM-PoseNet compared to the other approaches. From Table 2, we observe that Recurrent BIM-PoseNet fine-tuned with Gradmag-Syn-car images achieves the best localisation accuracy (1.62 m, 9.29${}^{\circ }$) amongst all approaches. Compared to [7,8], we see improvements in the accuracies for all the image renderings, with the exception of Syn-car images. Compared to [4] we notice improvements for all renderings except for Syn-pho-real images. In addition, Recurrent BIM-PoseNet achieves better results in contrast to VidLoc for the Syn-pho-real-tex, Gradmag-Syn-car and Syn-edge images. This improved accuracy of Recurrent BIM-PoseNet demonstrates the advantage of retaining the fully connected layers, as compared to VidLoc. This could be plausibly because the fully connected layers aid in the identification of the high-level structural information in the gradmag images, which the shallow layers of the network are incapable of. The improvement on rotation accuracy of Recurrent BIM-PoseNet is however apparent only for some of the renderings. DSAC++ [16] did not converge for any of the synthetic image renderings, in spite of having 2500 training images. Interestingly, for all approaches the performance of Gradmag-Syn-car and Syn-edge is quite similar and noticeably better than the other types of synthetic images.

**Figure 11:**
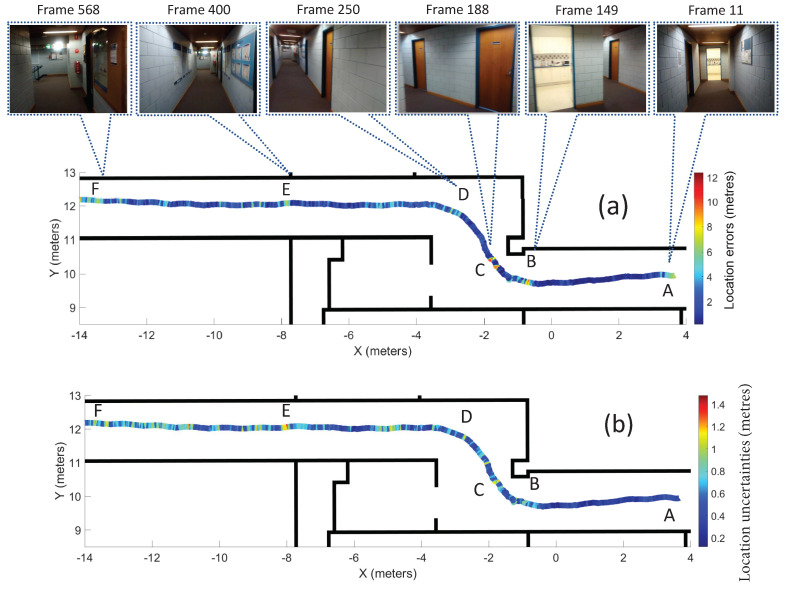
The distribution of the (**a**) location errors and (**b**) estimated location uncertainties along the trajectory for the network fine-tuned using Gradmag-Syn-car dataset. The first row shows some of the frames with large location errors near Points A–E. Table 3 shows the errors and estimated uncertainties of the frames.

Figure 11a shows the distribution of the errors and some of the frames that resulted in large errors along the trajectory (fine-tuned on Gradmag-Syn-car dataset), to identify error-prone areas. Table 3 shows the errors and estimated uncertainties for these frames. The large errors are likely the result of poor geometry of the scene, where the doors are far away from the camera, for example, near Point A. Other error sources include the motion blur that results in larger errors for the Points B, C and D of the trajectory. Additionally, the objects that are present in the images, but not in the 3D indoor model, such as the notice boards, poster and light flares (seen in Point E and F), are the other sources of errors. The errors near Point D in Figure 11a are reduced for Recurrent BIM-PoseNet (up to 6 m) as compared to [7] (up to 8 m) and [8] (up to 10 m).

### 4.6. Experiment 3: Modelling Uncertainty Using Synthetic Images

**Figure 12:**
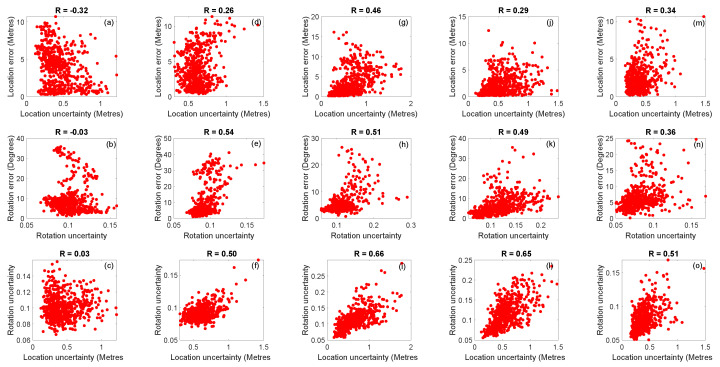
The uncertainty modelled by the network (**a**–**c**) fine-tuned using Syn-car and tested on real images, (**d**–**f**) fine-tuned using Syn-pho-real and tested on real images, (**g**–**i**) fine-tuned using Syn-pho-real-tex and tested on real images, (**j**–**l**) fine-tuned using Gradmag-Syn-car and tested on gradmag of real images, (**m**–**o**) fine-tuned using Syn-edge and tested on gradmag of real images. The first column shows the estimated location uncertainty vs. location error. The second column shows the estimated rotation uncertainty vs. rotation error, and the third column shows the estimated location uncertainty vs. estimated rotation uncertainty.

Figure 12 shows the correlation between the estimated uncertainties and the actual errors. Figure 12a–i show the (1) localisation errors vs. estimated location uncertainties, (2) rotation error vs. estimated rotation uncertainties and (3) estimated location uncertainties vs. estimated rotation uncertainties for the networks fine-tuned using the Syn-car, Syn-pho-real and Syn-pho-real-tex datasets. The network fine-tuned using Syn-pho-real-tex dataset shows the highest correlation for location errors vs. estimated location uncertainties, estimated location uncertainties vs. estimated rotation uncertainties and similar rotational errors vs. estimated rotation uncertainties, compared to the network fine-tuned using Syn-pho-real dataset. With the exception of the network fine-tuned using Syn-car dataset, there is an improvement in the correlation of the location and rotational uncertainties for Recurrent BIM-PoseNet compared to Bayesian BIM-PoseNet for Syn-pho-real (R=0.50vs.R=0.04) and Syn-pho-real-tex (R=0.66vs.R=0.33) datasets. However, the network fine-tuned using Syn-car dataset shows a negative correlation with location errors vs. estimated location uncertainties as well as with rotation errors vs. estimated rotation uncertainties. In addition, the correlation of the estimated location uncertainties vs. estimated rotation uncertainties are least as compared to the other datasets. Table 4 compares the correlation factor of Bayesian BIM-PoseNet with Recurrent BIM-PoseNet.

Figure 12j–o show the errors and the uncertainties for the networks fine-tuned using Gradmag-Syn-car and Syn-edge datasets. It is identified that the network fine-tuned using Gradmag-Syn-car dataset performs better as compared to the network fine-tuned using Syn-edge dataset for rotation error vs. estimated rotation uncertainty, and estimated rotation uncertainty vs. estimated location uncertainty. However, there is a slight decrease in the correlation for the location error vs. estimated location uncertainty. Compared to Bayesian BIM-PoseNet, the correlation of estimated location uncertainty vs. estimated rotational uncertainties improves for both the datasets and is summarised in Table 4. Moreover, the location error vs. estimated location uncertainty, and rotation error vs. estimated rotation uncertainty for the network fine-tuned using Gradmag-Syn-car dataset are better as compared to Bayesian BIM-PoseNet (Table 4).

Figure 11b shows the trend of the uncertainties modelled by the network fine-tuned on Gradmag-Syn-car dataset for the visualisation of the uncertain areas of the trajectory. It is observed that there is a good correlation between the errors (Figure 11a) and the uncertainties throughout the trajectory, except for Point A, where the errors are large, but the modelled uncertainties are low. Recurrent BIM-PoseNet is more confident in handling perceptual aliasing; for instance, near Point D in Figure 11. However, the uncertainty of camera pose estimation increases in the presence of artefacts such as notice boards, posters and light flares, and that explains the high uncertainties for Points E and F.

**Figure 13:**
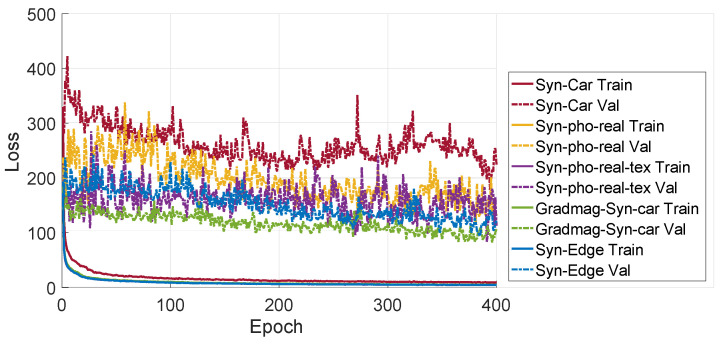
The training and validation losses for the networks fine-tuned with synthetic image sequences and validated with real images sequences, having a window size of 10.

Figure 13 shows the training and validation losses for the networks fine-tuned with a window of 10 synthetic images and validated with a window of 10 real images. It is observed that the validation losses of the networks are significantly higher as compared to the networks fine-tuned on real images (Figure 7). The higher validation losses might be a result of the differences between the synthetic and the real images (artefacts), and points towards a future research direction (explained in the conclusions).

### 4.7. Computation Times

Table 5 shows the fine-tuning times and test times of the networks for different window lengths. A Tesla P100 GPU (@1.32 GHz) having a memory of 12 GB and a Xeon (@2.20 GHz) CPU was used for fine-tuning the networks. A memory of 64 GB was allocated for the fine-tuning process and for testing a memory of 16 GB was allocated for all the networks. A batch size of 25 was used to fine-tune the networks, except for the network trained on a window of 15 images, where a batch size of 15 was used due to memory constraints. For reference, the test times of BIM-PoseNet and Bayesian BIM-PoseNet are 5 milliseconds and 67 milliseconds on a similar GPU, receptively.

As most of the smartphones are equipped with a GPU [37], Recurrent BIM-PoseNet can run in real-time on such devices, deeming it suitable for practical applications. However, there will be a lag in the camera pose estimation depending on the window length. For instance, while using a window length of ten images, there will be a lag of ten frames, which is feasible for real-time operations.

## 5. Conclusions

We propose a deep Bayesian recurrent CNN that, when fine-tuned using sequences of synthetic indoor images, can estimate the camera pose for a sequence of real images. The proposed approach eliminates any requirement of 3D reconstruction of the indoor space by SfM approaches. The results of the experiments suggest that an accuracy of 1.6 metres can be achieved by fine-tuning the proposed network using synthetic edge image sequences, that do not contain any colour or texture information, but only structural edges.

It is identified that a window of ten synthetic images is ideal for fine-tuning the proposed network for camera pose regression. The estimated camera poses results in a slightly smoother trajectory as compared to the existing approaches by exploiting the spatio-temporal information of the image sequences. Moreover, we show that the modelled uncertainty of the estimated camera poses is correlated with the errors.

The errors due to perceptual aliasing are reduced using image sequences as compared to the previous approaches, and as a result, the proposed network estimates the camera poses more accurately. The large errors are caused either due to the presence of motion blur or artefacts in the image, such as light flare or notice boards. Poor geometry of the scene is another error source where the image landmarks such as doors cover a small portion of the image being far away from the camera.

There is still some room for improvement in the current study that points towards interesting future directions. The semantic information of the BIM can be used to generate segmented images that can be used to fine-tune the proposed network, to reduce the effect of the artefacts. At the test time, semantically segmented real images [38] can be used.

## Figures and Tables

**Table 1:** The comparison of the median errors and inter-frame distances of different approaches.

Approach	Errors (Metres, Degrees)	Inter-Frame Distances (Metres)
PoseNet [1]	0.33 m, 1.85${}^{\circ }$	0.30 m
Bayesian PoseNet [2]	0.29 m, 1.53${}^{\circ }$	0.38 m
DSAC++ [16]	0.53 m, 0.61${}^{\circ }$	0.64 m
Walch et al. [4]	0.15 m, 1.62${}^{\circ }$	0.14 m
VidLoc [20]	0.16 m, 0.87${}^{\circ }$	0.10 m
Recurrent BIM-PoseNet (ours)	0.10 m, 0.83${}^{\circ }$	0.10 m

**Table 2:** The comparison of the errors of different approaches for the five synthetic image datasets being tested using real images. DSAC++ [16] did not converge for all the synthetic datasets in spite of containing 2500 training images.

Approach	Syn-Car	Syn-Pho-Real	Syn-Pho-Real-Tex	Gradmag-Syn-Car	Syn-Edge
BIM-PoseNet	6.25 m, 37.16${}^{\circ }$	5.99 m, 11.33${}^{\circ }$	3.06 m, 12.25${}^{\circ }$	2.63 m, 6.99${}^{\circ }$	1.88 m, 7.73${}^{\circ }$
Bayesian BIM-PoseNet	3.87 m, 8.38${}^{\circ }$	4.08 m, 25.03${}^{\circ }$	3.73 m, 13.53${}^{\circ }$	1.98 m, 7.33${}^{\circ }$	2.41 m, 12.53${}^{\circ }$
Walch et al. [4]	4.09 m, 22.28${}^{\circ }$	2.88 m, 15.31${}^{\circ }$	2.50 m, 11.99${}^{\circ }$	2.89 m, 19.22${}^{\circ }$	1.90 m, 12.42${}^{\circ }$
VidLoc [20]	3.04 m, 11.81${}^{\circ }$	2.78 m, 11.45${}^{\circ }$	2.73 m, 11.12${}^{\circ }$	2.60 m, 11.42${}^{\circ }$	2.30 m, 7.26${}^{\circ }$
Recurrent BIM-PoseNet	3.97 m, 15.20${}^{\circ }$	3.01 m, 8.50${}^{\circ }$	2.23 m, 8.31${}^{\circ }$	1.62 m, 9.29${}^{\circ }$	1.87 m, 11.15${}^{\circ }$

**Table 3:** The comparison of the errors and estimated uncertainties for the frames of Figure 11.

Frame	Location Error (Metre)	Location Uncertainties (Metre)	Rotation Error	Rotational Uncertainty
11	6.82	0.36	4.52${}^{\circ }$	0.09
149	7.60	0.41	2.85${}^{\circ }$	0.08
188	9.11	0.73	30.61${}^{\circ }$	0.16
250	5.92	0.68	9.67${}^{\circ }$	0.14
400	6.45	0.53	4.51${}^{\circ }$	0.09
568	6.91	0.53	15.16${}^{\circ }$	0.12

**Table 4:** The comparison of error and uncertainties for Recurrent BIM-PoseNet with Bayesian BIM-PoseNet.

Fine-Tuned on		Bayesian			Recurrent	
		BIM-PoseNet			BIM-PoseNet	
	$R_{\mathrm{LU}/\mathrm{LE}}$	$R_{\mathrm{RU}/\mathrm{RE}}$	$R_{\mathrm{LU}/\mathrm{RU}}$	$R_{\mathrm{LU}/\mathrm{LE}}$	$R_{\mathrm{RU}/\mathrm{RE}}$	$R_{\mathrm{LU}/\mathrm{RU}}$
Syn-car	0.12	0.31	0.34	−0.32	−0.03	0.03
Syn-pho-real	0.36	−0.01	0.04	0.26	0.54	0.50
Syn-pho-real-tex	0.33	0.53	0.33	0.46	0.51	0.66
Gradmag-Syn-car	0.42	0.50	0.59	0.29	0.49	0.65
Syn-edge	0.46	0.40	0.41	0.34	0.36	0.51

**Table 5:** The fine-tuning time and test times for the networks using different window lengths. The network with window length 1 is the implementation of BIM-PoseNet for comparing times.

Window Length	Fine-Tune Time (Hrs)	Test Time GPU (ms)	Test Time CPU (s)
1	1:44	12	0.13
3	4:34	16	0.40
5	7:07	35	0.67
10	12:43	52	0.94
15	23:48	108	2.12

## Supplementary Materials

The following are available online at https://melbourne.figshare.com/articles/UnimelbCorridorSynthetic_zip/10930457.
